# Cumulative ADHD medication use and risk of type 2 diabetes in adults: a Swedish Register study

**DOI:** 10.1136/bmjment-2024-301195

**Published:** 2024-09-25

**Authors:** Zihan Dong, Le Zhang, Lin Li, Shengxin Liu, Isabell Brikell, Ralf Kuja-Halkola, Brian M D’Onofrio, Agnieszka Butwicka, Soffia Gudbjornsdottir, Henrik Larsson, Zheng Chang, Ebba Du Rietz

**Affiliations:** 1 Department of Medical Epidemiology and Biostatistics, Karolinska Institute, Stockholm, Sweden; 2 Department of Biomedicine, Aarhus University, Aarhus, Denmark; 3 Department of Global Public Health and Primary Care, University of Bergen, Bergen, Norway; 4 Department of Psychological and Brain Sciences, Indiana University Bloomington, Bloomington, Indiana, USA; 5 Institute of Clinical Medicine, Faculty of Medicine, University of Oslo, Oslo, Norway; 6 Division of Mental Health Services R&D Department, Akershus University Hospital, Lørenskog, Norway; 7 Department of Biostatistics and Translational Medicine, Medical University of Lodz, Lodz, Poland; 8 Department of Molecular and Clinical Medicine, Sahlgrenska Academy, University of Gothenburg, Goteborg, Sweden; 9 Swedish National Diabetes Register, Centre of Registers Vastra Gotaland, Goteborg, Sweden; 10 School of Medical Sciences, Örebro University, Örebro, Sweden

**Keywords:** Data Interpretation, Statistical, Adult psychiatry, PSYCHIATRY

## Abstract

**Background:**

Little is known about the impact of cumulative attention-deficit/hyperactivity disorder (ADHD) medication use on the risk of type 2 diabetes (T2D).

**Objective:**

The objective is to examine the association between cumulative use of ADHD medication and risk of incident T2D.

**Methods:**

A nested case–control study was conducted in a national cohort of individuals aged 18–70 years with incident ADHD (n=138 778) between 2007 and 2020 through Swedish registers. Individuals with incident T2D after ADHD were selected as cases (n=2355) and matched with up to five controls (n=11 681) on age at baseline, sex and birth year. Conditional logistic regression models examined the association between cumulative duration of ADHD medication use and T2D.

**Findings:**

Compared with no use, a decreased risk of T2D was observed for those on cumulative use of ADHD medications up to 3 years (ORs: 0<duration≤1 year, 0.79 (95% CI, 0.69 to 0.91); 1<duration≤3 years, 0.80 (95% CI, 0.69 to 0.92); duration>3 years, 0.97 (95% CI, 0.84 to 1.12)). When investigating medication types separately, methylphenidate showed results similar to main analyses, lisdexamfetamine showed no association with T2D, whereas long-term (>3 years) use of atomoxetine was associated with an increased risk of T2D (OR: 1.44 (95% CI, 1.01 to 2.04)).

**Conclusion:**

Cumulative use of ADHD medication does not increase the risk for T2D, with the exception of long-term use of atomoxetine.

**Clinical implications:**

Findings suggest that clinicians should be aware of the potential risk of T2D associated with the cumulative use of atomoxetine among patients with ADHD; however, further replication is strongly needed.

**WHAT IS ALREADY KNOWN ON THIS TOPIC**

- Research suggests that long-term use of attention-deficit/hyperactivity disorder (ADHD) medication is associated with a significant increased risk of cardiovascular diseases.
- Previous studies have found mixed effects of ADHD medication on cardiometabolic risk factors associated with type 2 diabetes (T2D).
- The association of cumulative use of ADHD medication and T2D has never been studied.

**WHAT THIS STUDY ADDS**

- Our study found no strong evidence of elevated T2D risk, except for long-term use of atomoxetine.

**HOW THIS STUDY MIGHT AFFECT RESEARCH, PRACTICE OR POLICY**

- The findings suggest a need for heightened awareness of risk of T2D, particularly with long-term use of atomoxetine.

## Background

 Attention-deficit/hyperactivity disorder (ADHD) is a common neurodevelopmental condition characterised by impairing levels of inattention, hyperactivity and impulsivity that occurs in around 5% of children and adolescents and 2.5% of adults.^1^ Pharmacological therapy includes psychostimulants and non-psychostimulants. Psychostimulants act to boost norepinephrine and dopamine neurotransmission by blocking their reuptake and are recommended as first-line treatment for ADHD in many countries.^2^ This recommendation is based on the strong evidence from randomised controlled trials (RCTs), demonstrating the effectiveness of pharmacological therapy in reducing core ADHD symptoms.^3 4^ As for non-psychostimulants, atomoxetine is a selective norepinephrine reuptake inhibitor, and guanfacine is an alpha-2A adrenergic agonist, both leading to increased concentrations of norepinephrine in the prefrontal cortex.^2^ The use of ADHD medication, including long-term use, has increased greatly among adults in the past decades.^2^ A multicountry study showed that 30%–60% of patients with ADHD remained on medication for up to 5 years, considering treatment reinitiation.^5^ Furthermore, a recent study from Sweden demonstrated that long-term use of ADHD medications is associated with a moderately increased risk of cardiovascular diseases (CVDs).^6^ Similarly, a nationwide study in Denmark reported consistent findings, indicating that higher doses of ADHD treatment are linked to an elevated long-term cardiovascular risk in adults.^7^ As such, there is an increasing need to understand the long-term impact of ADHD medication on cardiometabolic health. Although emerging evidence has shown that individuals with ADHD have an increased risk of type 2 diabetes (T2D),^8^ a common metabolic disorder characterised by chronic hyperglycaemia, no previous research has investigated the impact of ADHD medication on the risk of T2D.

Prior studies indicate that ADHD medications may have short-term effects on cardiometabolic risk factors (eg, blood pressure, heart rate, weight) associated with T2D in adults.^9^ The direction of the association has, however, been mixed across different risk factors/medications studied. In terms of adverse effects, there has been consistent evidence from meta-analyses of RCTs showing minor but significant increases in blood pressure and heart rate from both psychostimulants and atomoxetine.^310^,^12^ It remains unknown whether the increase has cumulative effects over time and result in long-term adverse effects on T2D. Furthermore, one small clinical study has shown that psychostimulants may potentially have hyperglycaemic effects in young individuals.^13^ Additionally, selective norepinephrine reuptake inhibitors, of which atomoxetine belongs to, are similar to serotonin–norepinephrine reuptake inhibitors, which have been associated with weight gain.^14^

In terms of beneficial cardiometabolic effects, there is consistent evidence from RCTs that psychostimulants can result in weight loss in adults with ADHD,^3 15^ which is likely explained by decreased appetite from medication, especially in the early phases of treatment. Loss of appetite has also been reported for atomoxetine users^11 15 16^; however, only a few studies have showed weight loss after using atomoxetine.^11 16 17^ Moreover, a small clinical study of children and young adults showed tentative links between methylphenidate and improved lipid profile, as shown by significant reductions in total cholesterol, triglycerides and lipoprotein.^18^ Furthermore, ADHD medications are effective in improving ADHD symptoms (eg, disorganisation); it is therefore possible that ADHD medications could contribute to lowering the risk of T2D by reinforcing active decision-making towards healthy lifestyle choices and improved patterns of food intake.^19^

## Objective

Given the increasing use of ADHD medication worldwide, and its impact on cardiometabolic risk and protective factors associated with T2D, it is important to understand the association between ADHD medication use and risk of T2D. We conducted a nationwide population study to investigate the association between cumulative use of ADHD medications and the risk of T2D in adults and test whether the association differs by sex and age of T2D diagnosis.

## Methods

### Data sources

We used data from the following Swedish nationwide registers, linked via unique personal identification numbers^20^: The Total Population Register; National Patient Register^21^; Prescribed Drug Register^22^; Longitudinal Integrated Database for Health Insurance and Labour Market studies^23^; Swedish National Diabetes Register, see online supplemental material 1 for details.^24^

### Study design and cohort

A nested case–control design was used. We first selected a cohort of all individuals residing in Sweden aged 18–70 years with an incident diagnosis of ADHD (International Statistical Classification of Diseases and Related Health Problems (ICD-10) code F90) or incident dispensation of ADHD medication (ATC codes, see ‘Cumulative use of ADHD medications’ section), preceded by 18 months without a prescription for any ADHD medication,^6^ between 2007 and 2020. In Sweden, individuals exhibiting ADHD symptoms undergo a comprehensive neuropsychiatric evaluation before being diagnosed by a specialist.^25^ To identify the base cohort, we excluded individuals: (1) who had ADHD medication prescriptions for other indications^26^; (2) who had a T2D diagnosis before/at baseline (ICD-8/9 code: 250, ICD-10 code: E11); (3) who had emigration records before/at baseline; and (4) who died before/at baseline ((online supplemental material 1, supplementary figure 1). Cohort entry (baseline) was defined as the date of incident ADHD diagnosis or ADHD medication dispensation, whichever came first. The cohort was followed up until the date of a T2D diagnosis, death, emigration or the end of the study period, whichever came first.

Within this study cohort, T2D cases were identified if they had an incident diagnosis of T2D at 18 years or above during follow-up.^24^ The definition of T2D was based on ICD-10 code E11 diagnosis in the Swedish Register. The date of T2D diagnosis was assigned as the index date. Using incidence density sampling, each T2D case was randomly matched (on age at baseline, sex and birth year) with up to five controls without T2D from the ADHD study cohort. Controls were eligible if they were alive, living in Sweden and free of T2D at the time when their matched case received a T2D diagnosis. The index date for controls was the date of T2D diagnosis of the matched case.

### Cumulative use of ADHD medications

ADHD medications in the study included psychostimulants: methylphenidate, amphetamine, dexamphetamine and lisdexamfetamine, and non-psychostimulants: atomoxetine and guanfacine (ATC codes N06BA04/N06BA01/N06BA02/N06BA12/N06BA09/C02AC02). We studied overall ADHD medication use, as well as methylphenidate, lisdexamfetamine (approved for adults in Sweden only since 2015)^27^ and atomoxetine separately, because these medications have different mechanisms of action.^2^ We did not study the remaining ADHD medications separately, due to limited sample size. Number of days on ADHD medication use for each dispensation were derived from a validated algorithm, predicting treatment duration from free text in prescriptions.^26^ Cumulative duration of medication use was calculated by summing all treated days between the first prescription and the index date for each patient.

### Covariates

The following covariates were obtained at baseline: sociodemographic variables, including country of birth (Sweden, other) and highest educational level (primary/lower secondary, upper secondary, postsecondary or postgraduate), somatic comorbidities (CVDs, obesity, hyperlipidaemia, sleep disorders) and psychiatric comorbidities (anxiety disorders, autism spectrum disorder, bipolar disorder, conduct disorder, depressive disorder, eating disorders, intellectual disability, personality disorders, schizophrenia and substance use disorders) (online supplemental material 1, table S1).

### Statistical analyses

The cumulative duration of ADHD medication use was first categorised into four categories (no-use (reference group), 0<duration≤1, 1<duration≤3 and duration>3 years). Conditional logistic regression was used to estimate ORs for the associations between cumulative duration of ADHD medication use (overall, and for methylphenidate, lisdexamfetamine and atomoxetine separately) and incident T2D. Crude ORs were adjusted for all matching variables by design. Adjusted ORs additionally adjusted for covariates: sociodemographic, somatic and psychiatric comorbidities, and other ADHD medication use (when examining methylphenidate, lisdexamfetamine and atomoxetine separately). Cumulative duration of ADHD medication use was also modelled using a restricted cubic spline, to examine the potential non-linear relationship between ADHD medication use and risk of T2D.

In stratified analyses, associations were examined separately by sex and age at T2D diagnosis. In sensitivity analyses, we reran the main analyses restricting the sample to those who had ever used ADHD medications (methylphenidate, lisdexamfetamine and atomoxetine were also examined separately) to reduce unmeasured confounding between ADHD medication use and no-use groups. To further minimise the risk of misclassification of type 1 diabetes (T1D) into T2D, we conducted the main analyses while excluding T1D cases at baseline using the ICD-10 code E10 (those with ICD-8/9 codes 250 had already been excluded). Moreover, to reduce reverse causation, we reran the analysis by calculating the cumulative ADHD medication use from baseline to 3 months prior to the index date. The time window was selected because, in Sweden, routine psychiatric prescriptions are limited to maximum 3 months at a time.^28^

Data management and analyses were performed using SAS V.9.4. The report followed guidelines for Reporting of Studies Conducted Using Observational Routinely Collected Health Data – Pharmacoepidemiological Research.^29^

## Findings

After applying exclusion criteria, 125 628 individuals free from T2D were identified from 138 778 individuals with ADHD aged 18–70 years old. During a median follow-up of 5.7 (IQR: 2.6–9.0) years, 2359 individuals were diagnosed with T2D (online supplemental material 1, figure S1). Those with T2D were older and more prevalent with somatic and psychiatric comorbidities (except eating disorders) than those without T2D (online supplemental material 1, table S2). After applying incidence density sampling, 2355 individuals with T2D (1.7% of full ADHD cohort) and 11 681 matched controls were identified (online supplemental material 1, figure S1). The median age of this final sample was 41.1 (IQR: 32.2–48.8) years old at baseline, and follow-up time ranged from 0.01 to 13.9 years, with a median follow-up of 5.3 (IQR: 2.7–7.8) years. See table 1 for baseline characteristics. Individuals with T2D had higher rates of somatic and psychiatric comorbidities, and lower educational attainment compared with controls. A similar proportion of T2D cases (80.0%) and controls (84.3%) used at least one type of ADHD medication during follow-up, with methylphenidate being the most common, followed by atomoxetine (table 1).

**Table 1:** Characteristics of individuals with and without type 2 diabetes in the nested case–control sample

	T2D group (n=2355)	Non-T2D group (n=11 681)
Age at baseline, median (IQR), years	41.2(32.3, 49.0)	41.0(32.2, 48.8)
Follow-up time, median (IQR), years	5.3(2.7, 7.9)	5.3(2.7, 7.8)
Male, n (%)	1448 (61.5%)	7174 (61.4%)
Born in Sweden, n (%)	2022 (85.9%)	10 489 (89.8%)
Education*, n (%)		
Primary and lower secondary education	760 (32.3%)	3102 (26.6%)
Upper secondary education	1228 (52.1%)	5923 (50.7%)
Postsecondary and postgraduate education	320 (13.6%)	2549 (21.8%)
Unknown	47 (2.0%)	107 (0.9%)
Somatic comorbidities*, n (%)		
Obesity	235 (10.0%)	452 (3.9%)
CVDs	383 (16.3%)	1025 (8.8%)
Hyperlipidaemia	107 (4.5%)	160 (1.4%)
Sleep disorder	233 (9.9%)	635 (5.4%)
Psychiatric comorbidities*, n (%)		
Anxiety disorder	478 (20.3%)	1747 (15.0%)
Autism spectrum disorder	242 (10.3%)	883 (7.6%)
Bipolar disorder	277 (11.8%)	1073 (9.2%)
Conduct disorder	30 (1.3%)	85 (0.7%)
Depressive disorder	983 (41.7%)	4055 (34.7%)
Eating disorders	55 (2.3%)	222 (1.9%)
Intellectual disability	101 (4.3%)	232 (2.0%)
Personality disorders	466 (19.8%)	1708 (14.6%)
Schizophrenia	180 (7.6%)	559 (4.8%)
Substance use disorders	873 (37.1%)	3870 (33.1%)
Any ADHD medication use, n (%)	1883 (80.0%)	9849 (84.3%)
Methylphenidate use	1666 (70.7%)	8929 (76.4%)
Amphetamine use	7 (0.3%)	50 (0.4%)
Lisdexamfetamine use	413 (17.5%)	2318 (19.8%)
Dexamphetamine use	125 (5.3%)	858 (7.3%)
Atomoxetine use	600 (25.5%)	2813 (24.1%)
Guanfacine use	27 (1.1%)	112 (1.0%)

* Educational attainment and comorbidities for cases and controls were assessed at baseline.

ADHD, attention-deficit/hyperactivity disorder; CVD, cardiovascular disease; T2D, type 2 diabetes.

### Cumulative use of ADHD medications and risk of T2D

**Figure 1:**
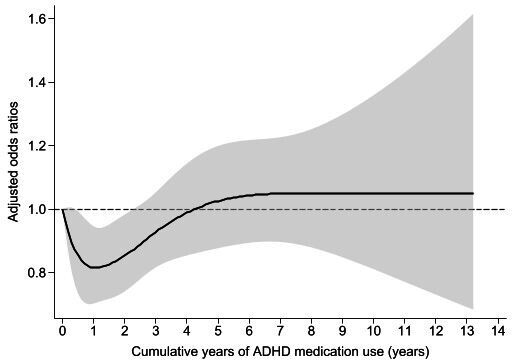
Association between cumulative duration of attention-deficit/hyperactivity disorder (ADHD) medication use and type 2 diabetes risk, when cumulative duration of ADHD medication use was modelled as continuous variable using restricted cubic spline. Number of knots: 5 (ie, 5th, 27.5th, 50th, 72.5th and 95th percentiles).

**Figure 2:**
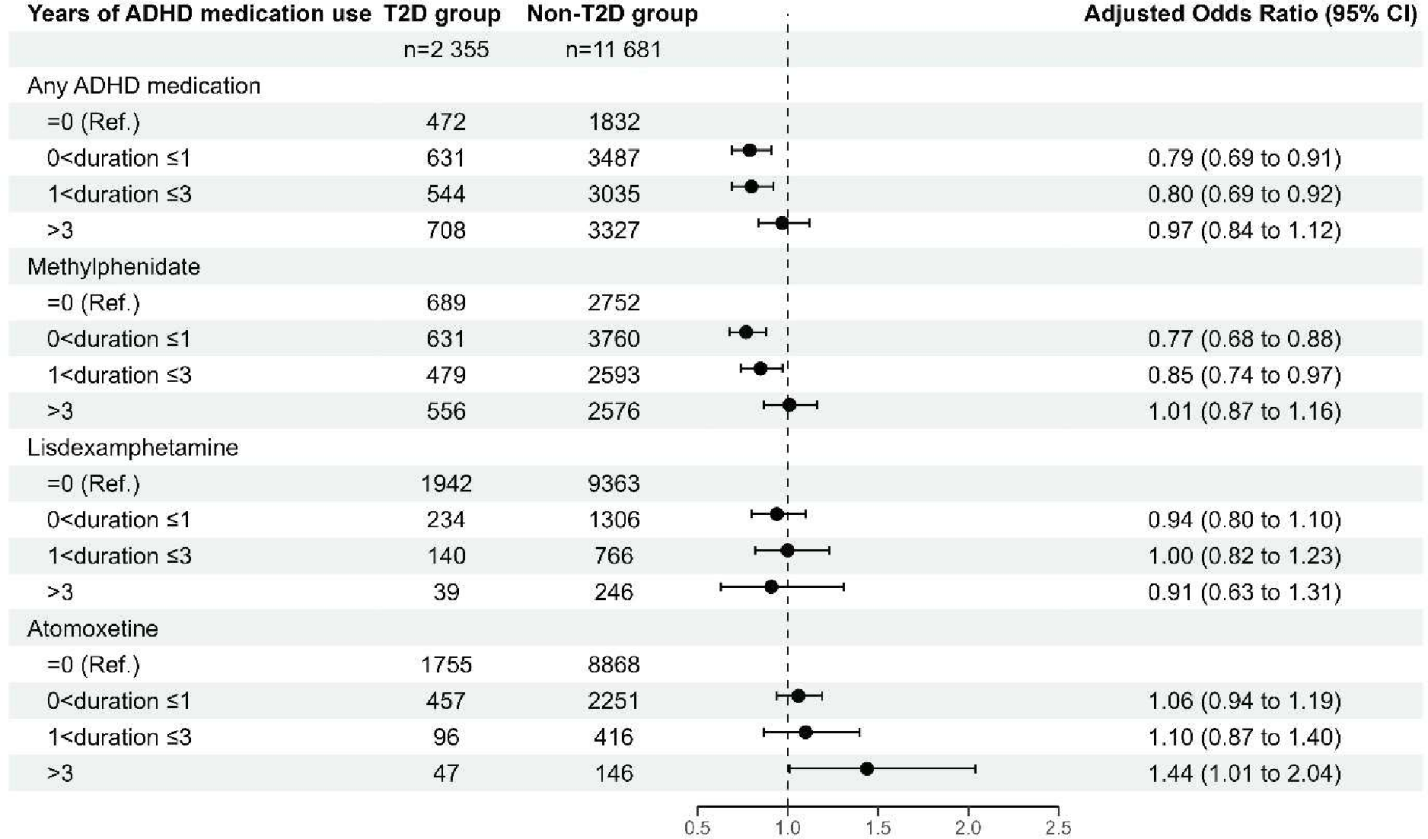
Association between cumulative duration of ADHD medication use and T2D risk, relative to no use of ADHD medication. Adjusted ORs are based on cases and controls matched on age, sex and time since baseline and adjusted for country of birth, highest educational level, somatic comorbidities, including cardiovascular disease, obesity, dyslipidaemia, sleep disorders and psychiatric comorbidities, including anxiety disorders, autism spectrum disorder, bipolar disorder, conduct disorder, depressive disorder, eating disorders, intellectual disability, personality disorders, schizophrenia and substance use disorders. ADHD, attention-deficit/hyperactivity disorder; T2D, type 2 diabetes.

The model using restricted cubic spline suggested a potential non-linear risk of T2D with longer duration of ADHD medication use (figure 1). The risk of T2D was statistically significantly lower for individuals who had been using ADHD medications for up to 3 years, compared with non-users, while individuals with longer durations of medication use did not show a significantly different risk of T2D (adjusted ORs: 0<duration≤1 year, 0.79 (95% CI, 0.69 to 0.91), p<0.01; 1<duration≤3 years, 0.80 (95% CI, 0.69 to 0.92), p<0.01; duration>3 years, 0.97 (95% CI, 0.84 to 1.12), p=0.69; figure 2). The crude model showed overall similar results, but with stronger associations and lower p values (online supplemental material 1, table S3).

When analysing specific types of ADHD medication, use of methylphenidate for less than 3 years was associated with a statistically significantly lower risk of T2D, compared with no use of methylphenidate (adjusted ORs: 0<duration≤1 year, 0.77 (95% CI, 0.68 to 0.88), p<0.0001; 1<duration≤3 years, 0.85 (95% CI, 0.74 to 0.97), p=0.02). For those who had been using methylphenidate for longer than 3 years, there was no statistically significant difference in the risk of T2D (adjusted OR: duration>3 years, 1.01 (95% CI, 0.87 to 1.16), p=0.95; figure 2). We found no statistically significant associations between cumulative lisdexamfetamine use and risk of T2D relative to no use of lisdexamfetamine (figure 2). Long-term use of atomoxetine was associated with increased risk of T2D, with statistically significant higher risk in those who used atomoxetine for longer than 3 years (adjusted ORs: 0<duration≤1 year, 1.06 (95% CI, 0.94 to 1.19), p=0.37; 1<duration≤3 years, 1.10 (95% CI, 0.87 to 1.40), p=0.43; duration>3 years, 1.44 (95% CI, 1.01 to 2.04), p=0.04; figure 2).

### Analyses stratified by sex and age at T2D diagnosis

**Figure 3:**
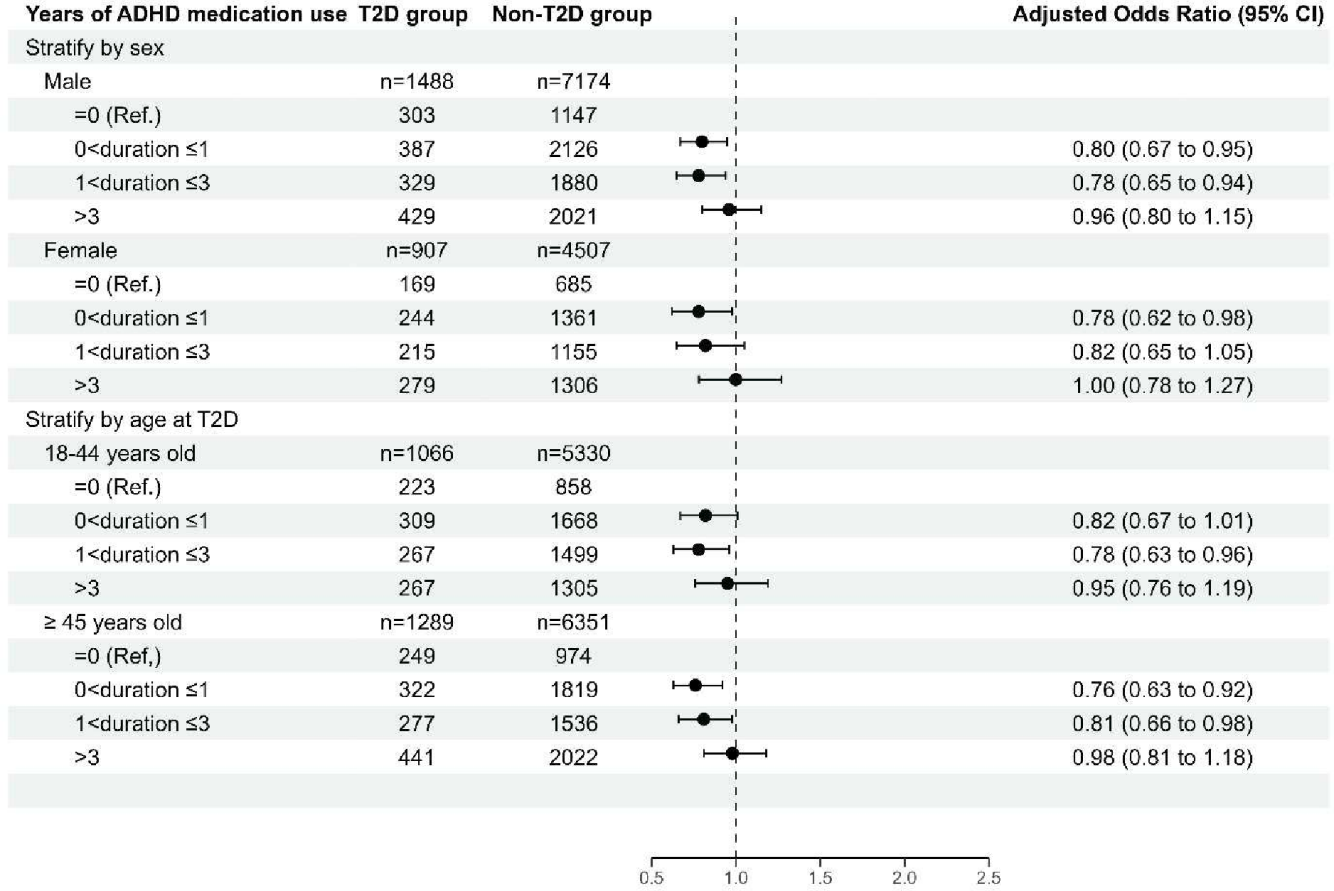
Association between cumulative duration of ADHD medication use and T2D risk, relative to no use of ADHD medication, stratified by sex and age group at T2D diagnosis. Adjusted ORs are based on cases and controls matched on age, sex and time since baseline and adjusted for country of birth, highest educational level, somatic comorbidities, including cardiovascular disease, obesity, dyslipidaemia, sleep disorders and psychiatric comorbidities, including anxiety disorders, autism spectrum disorder, bipolar disorder, conduct disorder, depressive disorder, eating disorders, intellectual disability, personality disorders, schizophrenia and substance use disorders. ADHD, attention-deficit/hyperactivity disorder; T2D, type 2 diabetes.

The overall results were similar across women and men, showing that cumulative ADHD medication use was associated with decreased risk for T2D compared with no medication use. We also found that cumulative ADHD medication use was associated with decreased risk for T2D for both age groups (≤44 years old, ≥45 years old) compared with no use (figure 3, online supplemental material 1, table S4).

### Sensitivity analyses

First, when restricting the sample to individuals who have been prescribed ADHD medications and using the lowest exposure category (≤1 year) as a reference group, we found significantly increased risk of T2D for individuals with cumulative ADHD medication use for longer than 3 years (adjusted OR: 1.22 (95% CI, 1.06 to 1.40), p<0.01), but not for individuals with cumulative ADHD medication use for only one to 3 years (adjusted OR: 1.00 (95% CI, 0.87 to 1.14), p=0.95; online supplemental material 1, table S5). When examining different types of ADHD medications, we found that methylphenidate and atomoxetine used for longer than 3 years were significantly associated with greater risk of T2D compared with the lowest exposure category (duration≤1 year) (methylphenidate: adjusted OR: 1.28 (95% CI, 1.10 to 1.48), p<0.01; atomoxetine: adjusted OR: 1.75 (95% CI, 1.00 to 3.04), p<0.05; online supplemental material 1, table S5). We found no statistically significant associations between cumulative lisdexamfetamine use and risk of T2D relative to the lowest exposure category (duration≤1 year) (online supplemental material 1, table S5). Second, when we further excluded T1D cases at baseline (n=70), the results were consistent with the main analyses (adjusted ORs: 0<duration≤1 year, 0.75 (95% CI, 0.65 to 0.86), p<0.0001; 1<duration≤3 years, 0.78 (95% CI, 0.67 to 0.90), p<0.01 duration>3 years, 0.94 (95% CI, 0.82 to 1.09), p=0.44; online supplemental material 1, supplementary table S6). Third, when calculating cumulative ADHD medication use from baseline to 3 months prior to the index date, the results were overall consistent with the main analyses for any ADHD medication use and different medication types (online supplemental material 1, table S7).

## Discussion

This is the first study to investigate the association between short-term and long-term use of ADHD medication and risk of T2D. Overall, we found no strong evidence that long-term use of ADHD medication increased the risk of T2D, if anything, a reduced risk was found for up to 3 years of cumulative use. These findings were consistent for methylphenidate, whereas there was no statistically significant association between lisdexamfetamine use and T2D, and there were tentative trends to suggest a minor increased risk of long-term atomoxetine use (>3 years) on T2D. It is important to note that the absolute risk of T2D is still minor (1.7% in the ADHD cohort). Future replication of study findings using different data sources and study designs is needed before firm conclusions are made about the potential risks or protective effects of ADHD medication on T2D.

We found a decreased risk of T2D associated with the use of ADHD medication (mainly explained by methylphenidate) of durations up to 3 years. This may be explained by the short-term effects (within the first year following medication initiation) of ADHD medication on the loss of appetite, decreased body weight and healthier lifestyle due to the management of ADHD symptoms.^3 11 15^ We can, however, not rule out the possibility that long-term ADHD medication use may still increase the risk of T2D, given the effect of ADHD medication on certain cardiometabolic risk factors (eg, high blood pressure),^310^,^12^ that may be associated with the risk of T2D. In line with this, our results suggested that the decreased risk of T2D associated with ADHD medication use did not persist for longer-term use (>3 years) of medication.

Sensitivity analyses showed that when restricting analyses to ever users of ADHD medication, the risk of T2D significantly increased with long-term medication use (>3 years). It is, however, possible that this increased risk for long-term ADHD medication use is driven by the change in the reference group (from no use to <1 year use), where taking ADHD medication for up to 1 year showed a lower risk of T2D relative to no use. As the individuals on long-term ADHD medication use (>3 years) did not show an increased risk for T2D compared with individuals who were never on medication, it does not seem to pose as particularly unsafe. Furthermore, the analysis with cumulative ADHD medication use calculating from baseline to 3 months prior to index date showed consistent results with the main analysis. In addition, the study examined the cumulative use of ADHD medication over time prior to T2D by study design. Therefore, people who were prescribed with stimulants were not likely to be those who were preselected for the absence of T2D. Future studies are needed to determine the effect of long-term use of ADHD medication on T2D, ideally using randomised control trials.

When we studied the different types of ADHD medications separately, we found a tentative increased risk of T2D with long-term cumulative use of atomoxetine (>3 years). In line with our findings, a meta-analysis showed that atomoxetine had stronger adverse effects on blood pressure and heart rate than methylphenidate, although this was only based on short-term medication use (<6 months) in children and adolescent.^10^ On the other hand, studies have also reported that atomoxetine is linked with weight loss in early treatment stages,^11 16^ although one study found that weight loss appeared to plateau after 1 year treatment,^30^ and the decreased weight might be related with weight at baseline.^11^ Confounding by indication might also explain the increased risk of T2D with atomoxetine use, given atomoxetine is not the first-line treatment of ADHD and usually prescribed when psychostimulants are not effective in Sweden. However, it is reassuring that the results were consistent when we restricted the analyses to only atomoxetine users. Given the limited sample size of atomoxetine users, especially with few long-term users of atomoxetine (>1 year), and borderline CI for the increased risk of T2D, long-term use of atomoxetine and the risk of T2D deserves further investigations.

If replicated, the findings of this study suggest that there may be a need for greater awareness of T2D risk among individuals with ADHD taking atomoxetine and that the use of atomoxetine should be used with caution. While our results cannot determine whether atomoxetine causes an increased risk of T2D, they suggest that individuals using atomoxetine for a longer period of time could be at potential risk of T2D. These individuals with ADHD may therefore need closer monitoring of glycaemic control and metabolic abnormalities.

### Strengths and limitations

This is the first nationwide population-based study investigating the risk of T2D associated with short-term and long-term use of ADHD medication. The use of Swedish registers made it a comprehensive and representative data source for the Swedish population. Using a validated algorithm for the prediction of treated days with ADHD medication from free-text medical records, we were able to derive a measure of medication use accounting for both quantity and dose over extended periods of time. We were also able to exclude the prescriptions for other indications than ADHD, which would have introduced bias. Furthermore, data were collected prospectively, so recall bias was low.

Our study also has limitations. First, we could not account for non-adherence to the treatment regimen. If misclassification of treated periods would be non-differential across cases and controls, our observed ORs would be conservative estimates. Second, even though several confounders were adjusted for, there may still have been residual confounding. When we compared the comorbidities at baseline among atomoxetine, methylphenidate and lisdexamfetamine users; however, these were similar, which indicates that the different associations between use of specific medications and T2D were not largely confounded (online supplemental material 1). Third, we had relatively limited sample sizes for analysing different medication types and for stratified samples. Studies with a larger sample size and longer follow-up are needed to replicate the results of subgroup estimates in the present study. Fourth, comorbidities were identified using the National Patient Register, and therefore may reflect severe patients in specialist care.

### Clinical implications

This nationwide register study with longitudinal follow-up showed no strong evidence that long-term ADHD medication use increased the risk of T2D, rather on the contrary, a reduced risk was found for up to 3 years of cumulative use, compared with individuals not using ADHD medication. However, long-term cumulative use of atomoxetine specifically showed a small but significant putative association with a higher risk for T2D. Future studies are needed to replicate and confirm our findings on the effect of ADHD medication use on T2D before firm conclusions can be made based on the results. It may, however, be beneficial for clinicians to be aware of this increased risk of T2D among ADHD patients using atomoxetine and to carefully consider the potential risks and benefits of ADHD medication when making treatment decisions.

## Supplementary material

10.1136/bmjment-2024-301195 online supplemental material 1

## Data Availability

Data may be obtained from a third party and are not publicly available.
