# Supplementary material for: Cumulative ADHD medication use and risk of type 2 diabetes in adults: a Swedish Register study
Source: BMJ Ment Health. 2024 Sep 25;27(1):e301195. doi: 10.1136/bmjment-2024-301195 (PMC11425947; doi:10.1136/bmjment-2024-301195)
Supplement: online supplemental material 1 [file bmjment-27-1-s001.pdf]

Supplementary Materials

Supplementary Methods. Information about Swedish registers

Supplementary Figure 1. Flow Chart of Selection of Case Patients with type 2 diabetes and Matched Controls

Supplementary Table 1. Anatomical Therapeutic Chemical (ATC) codes and International Classification of Diseases (ICD) Revisions codes used to define variables

Supplementary Table 2. Characteristics of individuals with and without type 2 diabetes in the overall cohort

Supplementary Table 3. Association between cumulative duration of ADHD medication use and type 2 diabetes risk, relative to non-users of ADHD medication

Supplementary Table 4. Stratified analysis - Association between cumulative duration of ADHD medication use and type 2 diabetes risk, relative to non-users of ADHD medication

Supplementary Table 5. Sensitivity analysis - Association between cumulative duration of ADHD medication use and type 2 diabetes risk, among ever-users of ADHD medication

Supplementary Table 6. Sensitivity analysis - Association between cumulative duration of ADHD medication use and type 2 diabetes risk, relative to non-users of ADHD medication, when further excluding individuals with type 1 diabetes (ICD-10 code: E10) at baseline

Supplementary Table 7. Sensitivity analysis - Association between cumulative duration of ADHD medication use and type 2 diabetes risk, relative to non-users of ADHD medication, when restrict exposure window between baseline to 3 months prior to index date

Supplementary Table 8. Characteristics of individuals with and without type 2 diabetes stratified by type of medication in the nested case-control sample

References

Supplementary Method. Information about Swedish registers

We used data from the following Swedish nationwide registers, linked via unique personal identification numbers:[1] The Total Population Register covering demographic information since 1968 and migration information since 1969; National Patient Register, including data on hospital and outpatient admissions since 1973 and 2001, respectively, according to the International Classification of Diseases (ICD) since 1969;[2] Prescribed Drug Register, covering all medication dispensations since 2005, using the Anatomic Therapeutic Chemical (ATC) code;[3] Longitudinal Integrated Database for Health Insurance and Labour Market studies, containing labour market, educational and social sector data;[4] Swedish National Diabetes Register, established in 1996, covering primary and secondary diabetes care in Sweden.[5]

Supplementary Figure 1. Flow chart of selection of case patients with type 2 diabetes and matched controls

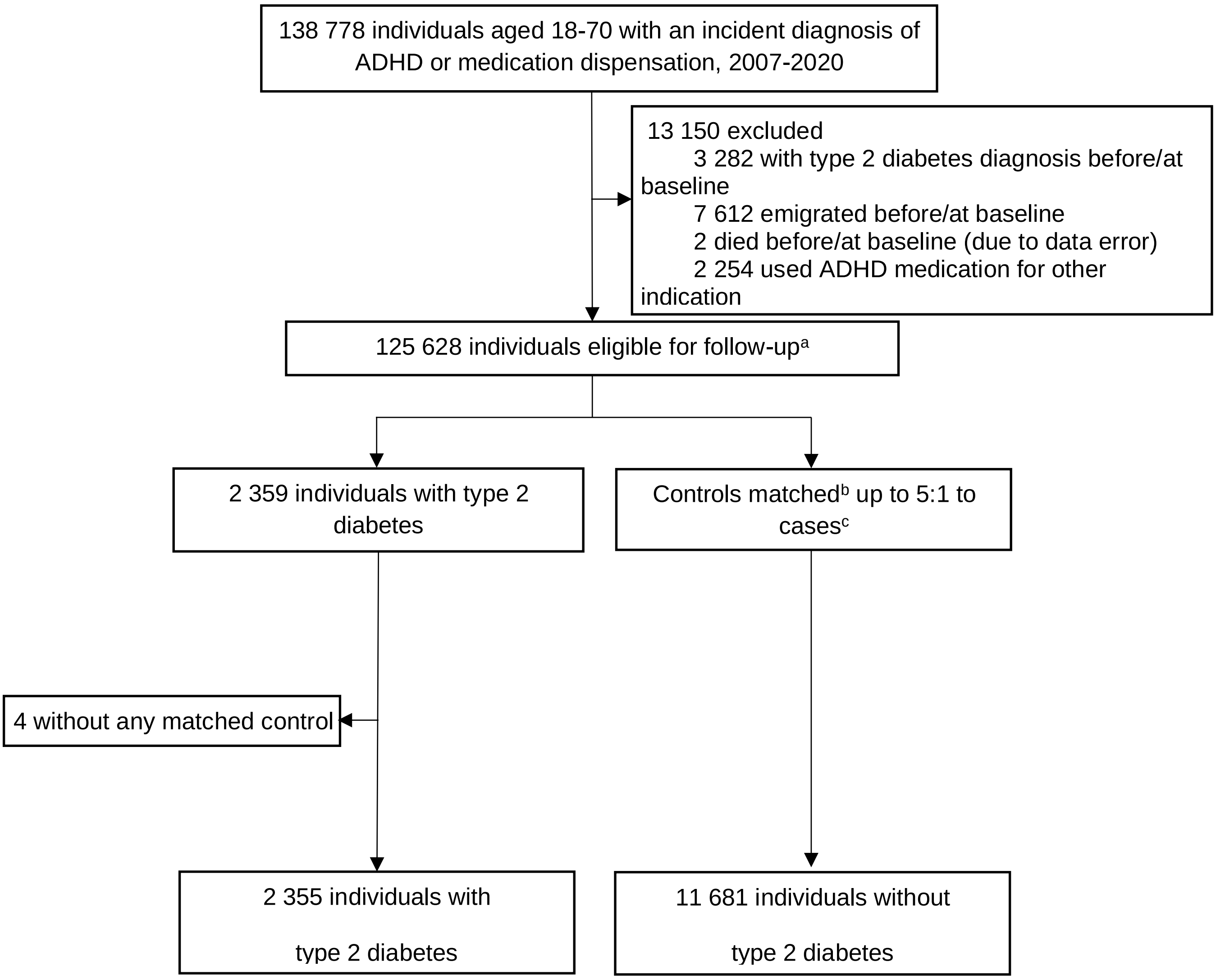

[a]Follow-up was defined as from first ADHD diagnosis/ADHD medication dispensation, until the earliest date of a T2D diagnosis, death, emigration, or the end date of the study, whichever came first

[b]The matching process employed incidence density sampling within a nested case-control design.[6]

[c]There are 36 cases had less than 5 controls

Supplementary Table 1. Anatomical Therapeutic Chemical (ATC) codes and International Classification of Diseases (ICD) Revisions codes used to define variables

	ICD codes from National Patient Register			ATC codes
	**ICD-8 Codes**	**ICD-9 Codes**	**ICD-10 Codes**	
Attention-deficit/hyperactivity disorder	-	-	F90	N06BA04, N06BA01, N06BA02, N06BA12, N06BA09, C02AC02
Type 2 diabetes mellitus	250	250	E11	
Type 1 diabetes mellitus	250	250	E10[a]	-
Obesity	277	278.A, 278.B	E65, E66	-
Any cardiovascular diseases	39-43, 440, 444, 445, 450-453, 458	390-430, 440, 444, 445	I0-I6, I70, I730, I74, I75	-
Hyperlipidemia	279	272	E78	C10
Sleep disorders	306.40	307E, 780F	G47	-
Anxiety disorder	300.0	300.00,300.02	F40-F41	-
Autism spectrum disorder	-	299	F84	-
Bipolar disorder	296.1, 296.3, 296.8	296A/ C/D/E/W	F30-F31	-
Conduct disorder	-	312	F91	-
Depressive disorder	296.2, 298.0,300.4	296B,300E	F32-33	-
Eating disorders	306.5x	307.5	F50	-
Intellectual disability	310-315	317-319	F7	-
Personality disability	301	301	F60-62, F69	-
Schizophrenia	295 except 295.7	295A-E/G/W/X	F2	-
Substance use disorder	291, 303, 304	291, 292, 304, 305A, 305X	F10-16, F18-F19	-

[a]Used only in sensitivity analysis

Supplementary Table 2. Characteristics of individuals with and without type 2 diabetes in the overall cohort

	T2D[a] group (N=2,359)	Non-T2D[a] group (N=123,269)
**Age at baseline, median [IQR[a]], years**	41.2 [32.3,49.1]	30.2 [23.6,39.8]
**Follow up time, median [IQR[a]], years**	5.3 [2.7,7.8]	5.7 [2.6,9.1]
**Male, No. (%)**	1,450 (61.5%)	63,244 (51.3%)
**Born in Sweden, No. (%)**	2,026 (85.9%)	111,089 (90.1%)
**Education[b], No. (%)**		
Primary and lower secondary education	761 (32.3%)	36,406 (29.5%)
Upper secondary education	1,228 (52.1%)	58,661 (47.6%)
Post-secondary and postgraduate education	323 (13.7%)	26,192 (21.3%)
Unknown	47 (2.0%)	2,010 (1.6%)
**Somatic comorbidities[b], No. (%)**		
Obesity	235 (10.0%)	6,263 (5.1%)
CVDs[a]	384 (16.3%)	7,167 (5.8%)
Hyperlipidemia	108 (4.6%)	792 (0.6%)
Sleep Disorder	234 (9.9%)	7,263 (5.9%)
**Psychiatric comorbidities[b], No. (%)**		
Anxiety disorder	478 (20.3%)	20,574 (16.7%)
Autism spectrum disorder	242 (10.3%)	12,194 (9.9%)
Bipolar disorder	277 (11.7%)	10,093 (8.2%)
Conduct disorder	30 (1.3%)	1,342 (1.1%)
Depressive disorder	985 (41.8%)	44,200 (35.9%)
Eating disorders	55 (2.3%)	4,561 (3.7%)
Intellectual disability	101 (4.3%)	2,821 (2.3%)
Personality disorders	467 (19.8%)	13,735 (11.1%)

	T2D[a] group (N=2,359)	Non-T2D[a] group (N=123,269)
Schizophrenia	181 (7.7%)	4,401 (3.6%)
Substance use disorders	874 (37.1%)	32,633 (26.5%)
**Any ADHD[a] medication use, No. (%)**	1887 (80.0%)	103,755 (84.2%)
Methylphenidate use	1670 (70.8%)	90,217 (73.2%)
Amphetamine use	7 (0.3%)	422 (0.3%)
Lisdexamfetamine use	413 (17.5%)	47,749 (38.7%)
Dexamphetamine use	125 (5.3%)	13,442 (10.9%)
Atomoxetine use	601 (25.5%)	25,821 (21.0%)
Guanfacine use	27 (1.1%)	3,270 (2.7%)

[a]Abbreviations: ADHD, attention-deficit/hyperactivity disorder; IQR, interquartile range; T2D, type 2 diabetes; CVD, cardiovascular disease
[b]Educational attainment and comorbidities for cases and controls were assessed at baseline

Supplementary Table 3. Association between cumulative duration of ADHD medication use and type 2 diabetes risk, relative to non-users of ADHD medication

Years of ADHD medication use	type 2 diabetes group (n=2 355)	Non-type 2 diabetes group (n=11 681)	Crude ORs	P-value	Adjusted ORs (95% CI)[b]	P-value
Any ADHD medication						
0	472 (20.0%)	1832 (15.7%)	1		1	
0<duration ≤1	631 (26.8%)	3487 (29.9%)	0.70	**<0.0001**	0.79 (0.69-0.91)	**0.001**
1<duration ≤3	544 (23.1%)	3035 (26.0%)	0.69	**<0.0001**	0.80 (0.69-0.92)	**0.002**
>3	708 (30.1%)	3327 (28.5%)	0.84	**0.01**	0.97 (0.84-1.12)	0.69
Methylphenidate						
0	689 (29.3%)	2752 (23.6%)	1		1	
0<duration ≤1	631 (26.8%)	3760 (32.2%)	0.67	**<0.0001**	0.77 (0.68-0.88)	**<0.0001**
1<duration ≤3	479 (20.3%)	2593 (22.2%)	0.74	**<0.0001**	0.85 (0.74-0.97)	**0.02**
>3	556 (23.6%)	2576 (22.1%)	0.87	**0.04**	1.01 (0.87-1.16)	0.95
Lisdexamphetamine						
0	1942 (82.5%)	9363 (80.2%)	1		1	
0<duration ≤1	234 (9.9%)	1306 (11.2%)	0.85	**0.04**	0.94 (0.80-1.10)	0.43
1<duration ≤3	140 (5.9%)	766 (6.6%)	0.86	0.12	1.00 (0.82-1.23)	0.98
>3	39 (1.7%)	246 (2.1%)	0.75	0.10	0.91 (0.63-1.31)	0.62
Atomoxetine						
0	1755 (74.5%)	8868 (75.9%)	1		1	
0<duration ≤1	457 (19.4%)	2251 (19.3%)	1.03	0.59	1.06 (0.94-1.19)	0.37
1<duration ≤3	96 (4.1%)	416 (3.6%)	1.17	0.17	1.10 (0.87-1.40)	0.43
>3	47 (2.0%)	146 (1.3%)	1.63	**0.004**	1.44 (1.01-2.04)	**0.04**

[a]Crude ORs are based on cases and controls matched on age, sex, and time since baseline
[b]Adjusted ORs are based on cases and controls matched on age, sex, and time since baseline and adjusted for country of birth, highest educational level, somatic comorbidities, including cardiovascular disease, obesity, dyslipidemia, sleep disorders, and psychiatric comorbidities, including anxiety disorders, autism spectrum disorder, bipolar disorder, conduct disorder, depressive disorder, eating disorders, intellectual disability, personality disorders, schizophrenia, and substance use disorders.

Supplementary Table 4. Stratified analysis - Association between cumulative duration of ADHD medication use and type 2 diabetes risk, relative to non-users of ADHD medication

Years of ADHD medication use	type 2 diabetes group (n=2 355)	Non-type 2 diabetes group (n=11 681)	Crude ORs (95% CI)[a]	P-value	Adjusted ORs (95% CI)[b]	P-value
**Stratify by sex**						
Male	n=1448	n=7174				
0	303 (20.9%)	1147 (16.0%)	1		1	
0<duration ≤1	387 (26.7%)	2126 (29.6%)	0.69 (0.59-0.82)	**<0.0001**	0.80 (0.67-0.95)	**0.01**
1<duration ≤3	329 (22.7%)	1880 (26.2%)	0.66 (0.56-0.79)	**<0.0001**	0.78 (0.65-0.94)	**0.009**
>3	429 (29.6%)	2021 (28.2%)	0.82 (0.69-0.97)	**0.02**	0.96 (0.80-1.15)	0.67
Female	n=907	n=4507				
0	169 (18.6%)	685 (15.2%)	1		1	
0<duration ≤1	244 (26.9%)	1361 (30.2%)	0.72 (0.58-0.90)	**0.004**	0.78 (0.62-0.98)	**0.03**
1<duration ≤3	215 (23.7%)	1155 (25.6%)	0.75 (0.60-0.94)	**0.01**	0.82 (0.65-1.05)	0.11
>3	279 (30.8%)	1306 (29.0%)	0.89 (0.71-1.11)	0.30	1.00 (0.78-1.27)	0.97
**Stratify by age at type 2**						
18-44 years	n=1066	n=5330				
0	223 (20.9%)	858 (16.1%)	1		1	
0<duration ≤1	309 (29.0%)	1668 (31.3%)	0.71 (0.59-0.86)	**0.0005**	0.82 (0.67-1.01)	0.06
1<duration ≤3	267 (25.1%)	1499 (28.1%)	0.68 (0.56-0.83)	**0.0002**	0.78 (0.63-0.96)	**0.02**
>3	267 (25.1%)	1305 (24.5%)	0.79 (0.64-0.98)	**0.03**	0.95 (0.76-1.19)	0.67
≥ 45 years	n=1289	n=6351				
0	249 (19.3%)	974 (15.3%)	1		1	
0<duration ≤1	322 (25.0%)	1819 (28.6%)	0.70 (0.58-0.84)	**0.0001**	0.76 (0.63-0.92)	**0.005**
1<duration ≤3	277 (21.5%)	1536 (24.2%)	0.71 (0.58-0.85)	**0.0003**	0.81 (0.66-0.98)	**0.03**
>3	441 (34.2%)	2022 (31.8%)	0.87 (0.73-1.05)	0.15	0.98 (0.81-1.18)	0.79

[a]Crude ORs are based on cases and controls matched on age, sex, and time since baseline
[b]Adjusted ORs are based on cases and controls matched on age, sex, and time since baseline and adjusted for country of birth, highest educational level, somatic comorbidities, including cardiovascular disease, obesity, dyslipidemia, sleep disorders, and psychiatric comorbidities, including anxiety disorders, autism spectrum disorder, bipolar disorder, conduct disorder, depressive disorder, eating disorders, intellectual disability, personality disorders, schizophrenia, and substance use disorders.

Supplementary Table 5. Sensitivity analysis - Association between cumulative duration of ADHD medication use and type 2 diabetes risk, among ever-users of ADHD medication

Years of ADHD medication use	type 2 diabetes group	Non-type 2 diabetes group	Crude ORs (95% CI)[a]	P-value	Adjusted ORs (95% CI)[b]	P-value
**Ever users**	n=1883	n=9849				
0<duration ≤1	631 (33.5%)	3487 (35.4%)	1		1	
1<duration ≤3	544 (28.9%)	3035 (30.8%)	0.98 (0.87-1.13)	0.85	1.00 (0.87-1.14)	0.95
>3	708 (37.6%)	3327 (33.8%)	1.19 (1.04-1.36)	**0.01**	1.22 (1.06-1.40)	**0.006**
**Ever users** - Methylphenidate	n=1666	n=8929				
0<duration ≤1	631 (37.9%)	3760 (42.1%)	1		1	
1<duration ≤3	479 (28.8%)	2593 (29.0%)	1.11 (0.97-1.27)	0.14	1.09 (0.94-1.26)	0.25
>3	556 (33.4%)	2576 (28.9%)	1.28 (1.11-1.48)	**0.0007**	1.28 (1.10-1.48)	**0.001**
**Ever users** - Lisdexamphetamine	n=413	n=2318				
0<duration ≤1	234 (56.7%)	1306 (56.3%)	1		1	
1<duration ≤3	140 (33.9%)	766 (33.1%)	1.05 (0.77-1.44)	0.76	1.16 (0.81-1.65)	0.41
>3	39 (9.4%)	246 (10.6%)	0.95 (0.57-1.57)	0.83	1.24 (0.70-2.20)	0.46
**Ever users** - Atomoxetine	n=600	n=2813				
0<duration ≤1	457 (76.2%)	2251 (80.0%)	1		1	
1<duration ≤3	96 (16.0%)	416 (14.8%)	1.15 (0.81-1.62)	0.44	1.17 (0.81-1.70)	0.40
>3	47 (7.8%)	146 (5.2%)	1.72 (1.04-2.86)	**0.04**	1.75 (1.00-3.04)	**0.048**

[a]Crude ORs are based on cases and controls matched on age, sex, and time since baseline
[b]Adjusted ORs are based on cases and controls matched on age, sex, and time since baseline and adjusted for country of birth, highest educational level, somatic comorbidities, including cardiovascular disease, obesity, dyslipidemia, sleep disorders, and psychiatric comorbidities, including anxiety disorders, autism spectrum disorder, bipolar disorder, conduct disorder, depressive disorder, eating disorders, intellectual disability, personality disorders, schizophrenia, and substance use disorders.

Supplementary Table 6. Sensitivity analysis - Association between cumulative duration of ADHD medication use and type 2 diabetes risk, relative to non-users of ADHD medication, when further excluding individuals with type 1 diabetes (ICD-10 code: E10) at baseline

Cumulative duration in years	type 2 diabetes group	Non-type 2 diabetes group	Crude ORs (95% CI)[a]	P-value	Adjusted ORs (95% CI)[b]	P-value
	n=2285	n=11331				
0	460 (20.1%)	1758 (15.5%)	1		1	
0<duration ≤1	602 (26.4%)	3386 (29.9%)	0.68 (0.59-0.78)	**<0.0001**	0.75 (0.65-0.86)	**<0.0001**
1<duration ≤3	525 (23.0%)	2916 (25.7%)	0.69 (0.60-0.79)	**<0.0001**	0.78 (0.67-0.90)	**0.0009**
>3	698 (30.6%)	3271 (28.9%)	0.83 (0.72-0.95)	**0.009**	0.94 (0.82-1.09)	0.44

[a]Crude ORs are based on cases and controls matched on age, sex, and time since baseline
[b]Adjusted ORs are based on cases and controls matched on age, sex, and time since baseline and adjusted for country of birth, highest educational level, somatic comorbidities, including cardiovascular disease, obesity, dyslipidemia, sleep disorders, and psychiatric comorbidities, including anxiety disorders, autism spectrum disorder, bipolar disorder, conduct disorder, depressive disorder, eating disorders, intellectual disability, personality disorders, schizophrenia, and substance use disorders.

Supplementary Table 7. Sensitivity analysis - Association between cumulative duration of ADHD medication use and type 2 diabetes risk, relative to non-users of ADHD medication, when restrict exposure window between baseline to 3 months prior to index date

Cumulative duration in years	type 2 diabetes group (n=2331)	Non-type 2 diabetes group (n=11562)	Crude ORs (95% CI)[a]	P-value	Adjusted ORs (95% CI)[b]	P-value
Any ADHD medication						
0	471 (20.2%)	1828 (15.8%)	1		1	
0<duration ≤1	648 (27.8%)	3581 (31.0%)	0.70 (0.61-0.80)	**<0.0001**	0.78 (0.68-0.89)	**0.0003**
1<duration ≤3	535 (23.0%)	2997 (25.9%)	0.69 (0.60-0.79)	**<0.0001**	0.79 (0.68-0.91)	**0.001**
>3	677 (29.0%)	3156 (27.3%)	0.85 (0.74-0.97)	**0.02**	0.96 (0.83-1.11)	0.61
Methylphenidate						
0	689 (29.6%)	2723 (23.6%)	1		1	
0<duration ≤1	638 (27.4%)	3800 (32.9%)	0.66 (0.59-0.75)	**<0.0001**	0.75 (0.66-0.85)	**<0.0001**
1<duration ≤3	472 (20.3%)	2556 (22.1%)	0.72 (0.64-0.83)	**<0.0001**	0.81 (0.71-0.93)	**0.003**
>3	532 (22.8%)	2483 (21.5%)	0.85 (0.75-0.98)	**0.02**	0.97 (0.84-1.11)	0.62
Lisdexamphetamine						
0	1946 (83.5%)	9398 (81.3%)	1		1	
0<duration ≤1	225 (9.7%)	1261 (10.9%)	0.85 (0.73-0.99)	**0.04**	0.89 (0.76-1.05)	0.16
1<duration ≤3	126 (5.4%)	708 (6.1%)	0.84 (0.69-1.03)	0.09	0.90 (0.73-1.10)	0.30
>3	34 (1.5%)	195 (1.7%)	0.82 (0.56-1.20)	0.31	0.96 (0.65-1.41)	0.83
Atomoxetine						
0	1741 (74.7%)	8861 (76.6%)	1		1	
0<duration ≤1	455 (19.5%)	2162 (18.7%)	1.08 (0.96-1.21)	0.20	1.08 (0.96-1.21)	0.22
1<duration ≤3	92 (4.0%)	396 (3.4%)	1.19 (0.94-1.50)	0.15	1.15 (0.90-1.46)	0.26
>3	43 (1.8%)	143 (1.2%)	1.53 (1.08-2.16)	**0.02**	1.42 (0.99-2.03)	0.05

[a]Crude ORs are based on cases and controls matched on age, sex, and time since baseline
[b]Adjusted ORs are based on cases and controls matched on age, sex, and time since baseline and adjusted for country of birth, highest educational level, somatic comorbidities, including cardiovascular disease, obesity, dyslipidemia, sleep disorders, and psychiatric comorbidities, including anxiety disorders, autism spectrum disorder, bipolar disorder, conduct disorder, depressive disorder, eating disorders, intellectual disability, personality disorders, schizophrenia, and substance use disorders.

Supplementary Table 8. Characteristics of individuals with and without type 2 diabetes stratified by type of medication[a] in the nested case-control sample

	Atomoxetine users		Methylphenidate users		Lisdexamfetamine users	
	type 2 diabetes group (N=600)	Non-type 2 diabetes group (N=2,813)	type 2 diabetes group (N=1,666)	Non-type 2 diabetes group (N=8,929)	type 2 diabetes group (N=413)	Non-type 2 diabetes group (N=2,318)
**Age at baseline, median [IQR], years**	40.5 [31.9,48.2]	40.8 [31.7,48.2]	40.9 [32.5,48.0]	40.7 [32.2,48.0]	39.7 [31.7,48.8]	40.0 [32.3,47.3]
**Follow up time, median [IQR], years**	5.90 [3.27,8.44]	5.94 [3.44,8.43]	5.69 [3.25,8.14]	5.54 [3.00,8.01]	5.77 [3.28,8.19]	5.69 [3.11,8.35]
**Male**	340 (56.7%)	1738 (61.8%)	1020 (61.2%)	5454 (61.1%)	260 (63.0%)	1368 (59.0%)
**Born in Sweden**	530 (88.3%)	2530 (89.9%)	1448 (86.9%)	8076 (90.4%)	352 (85.2%)	2100 (90.6%)
**Education[a]**						
Primary and lower secondary education	205 (34.2%)	783 (27.8%)	530 (31.8%)	2295 (25.7%)	129 (31.2%)	532 (23.0%)
Upper secondary education	312 (52.0%)	1428 (50.8%)	877 (52.6%)	4517 (50.6%)	215 (52.1%)	1193 (51.5%)
Post-secondary and postgraduate education	72 (12.0%)	583 (20.7%)	235 (14.1%)	2049 (22.9%)	68 (16.5%)	583 (25.2%)
Unknown	11 (1.8%)	19 (0.7%)	24 (1.4%)	68 (0.8%)	1 (0.2%)	10 (0.4%)
**Somatic comorbidities**						
Obesity	54 (9.0%)	119 (4.2%)	159 (9.5%)	346 (3.9%)	47 (11.4%)	89 (3.8%)
CVDs	71 (11.8%)	226 (8.0%)	240 (14.4%)	698 (7.8%)	52 (12.6%)	166 (7.2%)
Hyperlipidemia	27 (4.5%)	35 (1.2%)	69 (4.1%)	117 (1.3%)	14 (3.4%)	34 (1.5%)
Sleep Disorder	56 (9.3%)	161 (5.7%)	163 (9.8%)	491 (5.5%)	53 (12.8%)	152 (6.6%)
**Psychiatric comorbidities**						
Anxiety disorder	124 (20.7%)	447 (15.9%)	334 (20.0%)	1273 (14.3%)	92 (22.3%)	360 (15.5%)
Autism spectrum disorder	53 (8.8%)	164 (5.8%)	143 (8.6%)	597 (6.7%)	34 (8.2%)	137 (5.9%)

	Atomoxetine users		Methylphenidate users		Lisdexamfetamine users	
	type 2 diabetes group (N=600)	Non-type 2 diabetes group (N=2,813)	type 2 diabetes group (N=1,666)	Non-type 2 diabetes group (N=8,929)	type 2 diabetes group (N=413)	Non-type 2 diabetes group (N=2,318)
Bipolar disorder	77 (12.8%)	278 (9.9%)	185 (11.1%)	759 (8.5%)	45 (10.9%)	202 (8.7%)
Conduct disorder	9 (1.5%)	25 (0.9%)	18 (1.1%)	61 (0.7%)	2 (0.5%)	23 (1.0%)
Depressive disorder	256 (42.7%)	985 (35.0%)	700 (42.0%)	3023 (33.9%)	190 (46.0%)	856 (36.9%)
Eating disorders	14 (2.3%)	64 (2.3%)	40 (2.4%)	169 (1.9%)	11 (2.7%)	57 (2.5%)
Intellectual disability	18 (3.0%)	47 (1.7%)	57 (3.4%)	133 (1.5%)	10 (2.4%)	21 (0.9%)
Personality disorders	126 (21.0%)	473 (16.8%)	327 (19.6%)	1197 (13.4%)	62 (15.0%)	291 (12.6%)
Schizophrenia	44 (7.3%)	146 (5.2%)	113 (6.8%)	390 (4.4%)	24 (5.8%)	89 (3.8%)
Substance use disorders	252 (42.0%)	1060 (37.7%)	647 (38.8%)	2861 (32.0%)	164 (39.7%)	767 (33.1%)
**Any ADHD medication use**	600 (100%)	2813 (100%)	1666 (100%)	8929 (100%)	413 (100%)	2318 (100%)
Methylphenidate use	426 (71.0%)	2122 (75.4%)	1666 (100%)	8929 (100%)	369 (89.3%)	2094 (90.3%)
Amphetamine use	1 (0.2%)	11 (0.4%)	3 (0.2%)	34 (0.4%)	1 (0.2%)	16 (0.7%)
Lisdexamfetamine use	134 (22.3%)	680 (24.2%)	369 (22.1%)	2094 (23.5%)	413 (100%)	2318 (100%)
Dexamphetamine use	53 (8.8%)	262 (9.3%)	111 (6.7%)	786 (8.8%)	93 (22.5%)	561 (24.2%)
Atomoxetine use	600 (100%)	2813 (100%)	426 (25.6%)	2122 (23.8%)	134 (32.4%)	680 (29.3%)
Guanfacine use	11 (1.8%)	44 (1.6%)	23 (1.4%)	103 (1.2%)	20 (4.8%)	65 (2.8%)

[a]Atomoxetine users, methylphenidate users and lisdexamfetamine users are not mutually exclusive

References

1 Ludvigsson JF, Otterblad-Olausson P, Pettersson BU, Ekbom A. The Swedish personal identity number: Possibilities and pitfalls in healthcare and medical research. *Eur J Epidemiol* 2009; **24**: 659-67. doi: 10.1007/s10654-009-9350-y.

2 Ludvigsson JF, Andersson E, Ekbom A, *et al.* External review and validation of the Swedish national inpatient register. *BMC Public Health* 2011; **11**: 450. doi: 10.1186/1471-2458-11-450.

3 Wettermark B, Hammar N, Fored CM, *et al.* The new Swedish Prescribed Drug Register--opportunities for pharmacoepidemiological research and experience from the first six months. *Pharmacoepidemiol Drug Saf* 2007; **16**: 726-35. doi: 10.1002/pds.1294.

4 Ludvigsson JF, Svedberg P, Olén O, Bruze G, Neovius M. The longitudinal integrated database for health insurance and labour market studies (LISA) and its use in medical research. *Eur J Epidemiol* 2019; **34**: 423-37. doi: 10.1007/s10654-019-00511-8.

5 Svensson AM, Eliasson B, Linder E, *et al. Nationwide Results 1996-2020*. Swedish National Diabetes Register, 2021. doi:10.18158/ryNUNVPiu.

6 Sedgwick P. Nested case-control studies. *BMJ* 2010; **340**: c2582. doi: 10.1136/bmj.c2582.
